# The Friendship Gap: Investigating gender differences in adolescent friendships and mental health

**DOI:** 10.1192/j.eurpsy.2024.742

**Published:** 2024-08-27

**Authors:** T. Manchanda, A. Stein, M. Fazel

**Affiliations:** ^1^Psychiatry, University of Oxford, Oxford, United Kingdom

## Abstract

**Introduction:**

Friendships are vital relationships throughout the lifespan, but become especially meaningful during adolescence. Adolescents between the ages of 10 and 18 name a friend as one of the most important people in their lives (Kiesner et al., 2004). Authentic social groups, defined as mutual social relationships that adolescents voluntarily engage in, are sources of support and companionship for adolescents, more than parents (Furman & Buhrmester, 1992). Past research shows adolescents turn to their friends most for mental health support in a crisis, yet less than half report finding the support helpful (Geulayov et al., 2022). Thus, it’s crucial to understand friendship dynamics of adolescents in order to address an appropriate intervention. Past literature has demonstrated gender differences in how adolescents approach friendships and social relationships (Lempers & Clark- Lempers, 1993).

**Objectives:**

I aim to investigate whether girls, boys, and gender non-binary individuals differ in their perceptions of friendship quality and friendship dynamics (i.e. social support seeking) and whether these differences have implications for their mental health outcomes. By studying gender differences in friendship quality and mental health, I hope to shed light on potential avenues for promoting inclusivity and positive mental health outcomes for both gender binary and gender non-binary adolescents.

**Methods:**

A cross-sectional survey (OxWell) was administered online to students across secondary schools and further education colleges in England to assess their self-reported friendship quality. The RCADS and WEMWBS scales were used to assess depression and anxiety symptomology, and well-being, respectively. The results from the survey were analysed in R.

**Results:**

Gender-binary and gender non-binary adolescents differed in friendship quality, friendship dynamics, mental health scores, and help-seeking behaviours. Gender non-binary adolescents had the worst mental health scores and reported lowest friendship quality compared to girls and boys. Boys had the best mental health when compared to girls and gender non-binary adolescents, and were more likely to perceive support provided by their friends as helpful. Surprisingly, gender non-binary adolescents reached out to their friends the most (when compared to girls and boys) for mental health support, despite having proportionally lower quality friendships, and were the least likely to find support received from friends helpful.

**Conclusions:**

This data presents evidence for the difference in social relationships across adolescents of all genders. It highlights the need for specialized and inclusive mental health support being made available for gender non-binary youth in England—a minoritized group in need of intervention. This data hopes to inform school-based friendship interventions targeted to improve friendships and mental health of gender non-binary youth.

**Disclosure of Interest:**

None Declared

